# Endothelial Function Responses to Nigella sativa (Black Seed) Supplementation: A Systematic Review and Meta-Analysis of Randomized Controlled Trials

**DOI:** 10.7759/cureus.61047

**Published:** 2024-05-25

**Authors:** Mahmoud Ali, Mariam Tantawi, Abdallah Hussien Kamel, Zeyad Firas Tameemi, Afnan A Rezk, Mahmoud Abdo, Rania Shaker Mostafa, Alaa Ramadan, Mohamed Elmasry, Eshak I Bahbah

**Affiliations:** 1 Internal Medicine, Al-Azhar University, Damietta, EGY; 2 Internal Medicine, Ain Shams University, Cairo, EGY; 3 Internal Medicine, Assuit University, Assuit, EGY; 4 Internal Medicine, Ulyanovsk State University, Ulyanovsk, RUS; 5 Epidemiology and Public Health, Alexandria University, Alexandria, EGY; 6 Clinical Pharmacy, School of Pharmaceutical Sciences, Universiti Sains Malaysia, Penang, MYS; 7 Internal Medicine, Cairo University, Cairo, EGY; 8 Medicine, South Valley University, Qena, EGY; 9 Surgery, Alexandria University, Alexandria, EGY; 10 Neurology, Al-Azhar University, Damietta, EGY

**Keywords:** systematic review and meta-analysis, adhesion molecules, vascular disorder, endothelial function, nigella sativa

## Abstract

*Nigella sativa* (NS), commonly known as black cumin or black seed, is a medicinal plant with a rich history of traditional use in various cultures. Recent research has shed light on its potential therapeutic properties, particularly its effects on endothelial markers involved in inflammatory processes. This systematic review and meta-analysis evaluated the endothelial function responses, including intercellular adhesion molecule (ICAM) and vascular cell adhesion molecule (VCAM), to NS supplementation. We systematically searched Medline via PubMed, Scopus, Web of Science, and Embase databases from inception until August 5, 2023. Comparative randomized controlled trials (RCTs) were included. Pairwise meta-analysis was conducted using RevMan version 5.4 for Windows. Pooled data were reported as mean difference (MD), with their 95% confidence interval (CI). The present meta-analysis included three RCTs, which included 146 patients. The pooled random-effect size showed no difference between the NS group and the control group in terms of ICAM (MD = -59.32, 95% CI: -137.18 to 18.54; p = 0.14) and VCAM (MD = -200.1, 95% CI: -429.9 to 29.69; p = 0.09). The pooled data were severely heterogeneous. In conclusion, NS supplementation does not have a significant impact on the endothelial function of patients with CVD or the risks of CVD. Further comparative RCTs with larger sample sizes and more diverse populations are needed to establish the efficacy and safety of NS in different clinical settings.

## Introduction and background

Endothelial function is critical in the context of cardiovascular diseases (CVD) as it plays a pivotal role in regulating vascular tone and blood flow and maintaining vascular homeostasis [1]. Impaired endothelial function is a key early marker of atherosclerosis and has been linked to the development and progression of various cardiovascular conditions, highlighting its importance for cardiovascular health and disease prevention [2]. Endothelial dysfunction is characterized by a reduction in the bioavailability of nitric oxide and an imbalance in the production of vasodilators and vasoconstrictors, leading to a pro-inflammatory and pro-thrombotic state [3]. This dysfunction is closely associated with increased expression of intercellular adhesion molecule-1 (ICAM-1) and vascular cell adhesion molecule-1 (VCAM-1), which are key mediators in the adhesion of leukocytes to the endothelium [4]. Elevated levels of ICAM-1 and VCAM-1 contribute to the initiation and progression of atherosclerosis by promoting the infiltration of inflammatory cells into the vascular wall, thereby exacerbating the inflammatory response and plaque formation [5].

Efforts to enhance endothelial function have received much attention in recent years [6,7]. One such option is to take natural supplements. *Nigella sativa* (NS), often known as black seed or black cumin, has been used in traditional medicine for ages for its numerous therapeutic effects. Recent research has focused on its possible involvement in enhancing endothelial function and lowering the risk of CVD [8,9]. NS is a flowering plant native to Southwest Asia that has been widely used in numerous civilizations as a spice and herbal cure [10]. It includes several bioactive chemicals that are thought to be responsible for its therapeutic benefits, including thymoquinone, thymohydroquinone, and dithymoquinone. These substances have antioxidant, anti-inflammatory, anti-hypertensive, and anti-thrombotic properties that may help enhance endothelial function [11].

Several investigations on the impact of NS supplementation on endothelial function have been conducted. Animal studies showed that taking black seed extract enhances endothelium-dependent vasodilation by increasing nitric oxide (NO) production and decreasing oxidative stress. It has also been proven to lower inflammatory markers and limit platelet aggregation, which contributes to enhanced endothelial function [12,13]. Recent investigations found that NS inhibits the expression of ICAM and VCAM molecules in endothelial cells. By reducing ICAM and VCAM expression, NS may help decrease immune cell adhesion and migration into inflamed tissues. This can potentially alleviate inflammation-associated symptoms and promote tissue healing [14,15].

Despite these encouraging findings from experimental studies, clinical data that supports the NS benefits on endothelial functions are limited. In addition, the recently published clinical trials showed conflicting results. For example, Tavakoli-Rouzbehani et al. showed a significant reduction in the levels of ICAM and VCAM after administrating NS [15]. However, Emamat et al., showed that the effect of NS and placebo on ICAM and VCAM was comparable, with no statistically significant difference [16]. Therefore, in this systematic review and meta-analysis, we aimed to evaluate the endothelial function responses of ICAM and VCAM to NS (black seed) supplementation.

## Review

Methods

The preparation of this manuscript was in line with the Preferred Reporting Items for Systematic Reviews and Meta-Analyses (PRISMA) statement [17].

Search strategy

A comprehensive search was executed on four key electronic databases, Medline via PubMed, Scopus, Web of Science, and EMBASE, from their inception until August 2023. Various combinations of the following keywords were used. (("Nigella sativa" OR "Black Cumin" OR "Kalonji") AND ("endothelial function" OR "oxidative stress" OR "Intracellular Adhesion Molecule" OR "Vascular Cell Adhesion Molecule" OR "anthropometric indices")). We employed MeSH terms to enhance the precision of our search. Additionally, references to the included studies were manually searched to detect other eligible studies.

Eligibility criteria

We included randomized controlled trials (RCTs) that recruited adult patients who received NS supplementation. Studies were deemed eligible if they included patients with at least one CVD risk factor or CVD. Only comparative RCTs, which compared NS to placebo, were included. The primary outcomes of interest were ICAM-1 and VCAM-1. We excluded studies that were reported in languages other than English, unpublished reports, theses, review articles, conference abstracts, and editorials. We excluded these studies to ensure the reliability and quality of the data, as they may lack rigorous peer review, standardized methodologies, or comprehensive detail necessary for robust meta-analytic conclusions.

Data extraction

Three independent authors extracted data using a standardized data extraction form. We retrieved the following information from the included study: study design, country, sample size, age, sex, weight, type of disease, inclusion criteria, exclusion criteria, aim, and key findings. Disagreements between authors were managed through consensus or consultation.

Risk of bias assessment

The revised Cochrane risk-of-bias tool for randomized trials (ROB2) was employed to assess the risk of bias in the included studies [18]. The ROB2 tool evaluates the following domains: bias arising from the randomization process, bias due to deviations from intended interventions, bias due to missing outcome data, bias in the measurement of the outcome, and bias in the selection of the reported result. Each domain was rated as "low risk," "high risk," or "some concerns." Disagreements between authors were managed through consensus.

Statistical analysis and heterogeneity assessment

We used RevMan software version 5.4 (Cochrane Collaboration, London, United Kingdom) for Windows for data analysis. Continuous data were pooled as a mean difference (MD) with a 95% confidence interval (CI) using random-effects models to account for the anticipated heterogeneity among the included studies. A p-value of less than 5% was considered statistically significant. The heterogeneity in the pooled analysis was assessed using visual inspection of the forest plots and I2 statistics. I2 values of 25%, 50%, and 75% were considered low, moderate, and high heterogeneity, respectively. A p-value of less than 0.05 was considered statistically significant. No subgroup analyses were performed due to the small number of included RCTs. Likewise, we did not assess the publication bias as the number of included studies was less than 10.

Results

**Figure 1:**
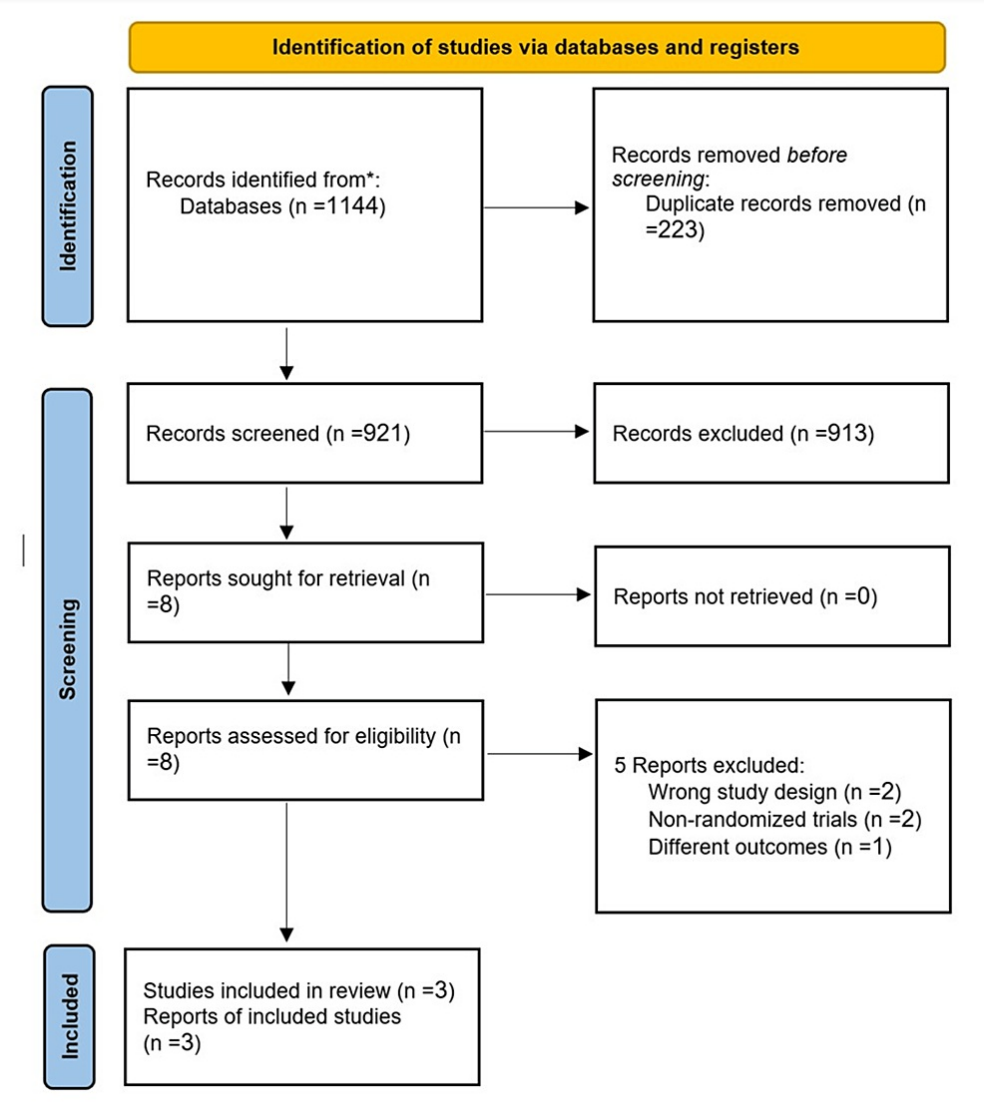
PRISMA Flowchart *Electronic databases such as Medline via PubMed, Scopus, Web of Science, and EMBASE. PRISMA: Preferred Reporting Items for Systematic Reviews and Meta-Analyses

Our search retrieved a total of 1144 articles. After removing the duplicates, 921 records were eligible for title and abstract screening. Eight studies entered the full-text screening. Finally, three studies were included in our meta-analysis. The selection process of the included studies is shown in Figure 1.

Characteristics of included studies and risk of bias

The populations studied within the three RCTs included patients with coronary artery disease (CAD) in two trials [15,16] and patients with Hashimoto's thyroiditis in one [14]. Included patients were predominantly having hypertension (HTN), diabetes mellitus (DM), dyslipidemia, and smoking status. The sample sizes were relatively small, ranging from 23 to 25 participants per group. There was a noticeable male predominance in the CAD studies (75-84%), while Hashimoto's thyroiditis study had only 15% male participants. Age ranges also varied, with the CAD studies having older participants (around 44-56 years) compared to the Hashimoto's study (around 34-36 years). The form and dosage of NS varied. Emamat et al. [16] used two capsules of 500 mg each, Tavakoli-Rouzbehani et al. [15] used 2 gm of NS oil, and Farhangi and Tajmiri [14] used 2 gm of NS powder. The duration of follow-up was almost two months across the included studies. The baseline and summary tables of the included studies are shown in Table 1.

**Table 1:** Baseline and summary of included studies CAD: Coronary artery disease; NS: *Nigella sativa*; P: Placebo; HTN: Hypertension; DM: diabetes mellitus; NR: Not reported; RCT: Randomized controlled trial

Study ID	Country	Study design	Sample Size (NS/P)	CAD or CAD risk	Age (NS/P)	Male (NS/P)	Weight (NS\P)	HTN (NS/P)	DM (NS/P)	Dyslipidemia (NS/P)	Smoker (NS/P)	NS dose	NS duration	Follow-up duration
Emamat et al. 2022 [16]	Iran	RCT	25	CAD	44.28 (11.29)	21 (84%)	73.63 (6.78)	11 (44%)	6 (24%)	14 (56%)	8 (32%)	2 capsules of 500 mg NS	30 minutes before bedtime	2 months
			25		42.80 (7.79)	21 (84%)	75.99 (9.77)	10 (40%)	6 (24%)	16 (64%)	7 (28%)			
Farhangi et al. 2020 [14]	Iran	RCT	23	CAD risk factor (autoimmune hypothyroidism)	35.7 (8.18)	6 (15%)	70.52 (12.27)	NR	NR	NR	NR	2 gm of NS powder	NR	8 weeks
			24		33.95 (8.72)	7 (15%)	69.63 (11.75)	NR	NR	NR	NR	2 gm of starch		8 weeks
Tavakoli-Rouzbehani et al. 2022 [15]	Iran	RCT	25	CAD	54.25 (1.55)	18 (75%)	77.48 (10.62)	19 (76%)	NR	NR	NR	2 gm of NS oil	NR	8 weeks
			24		55.92 (1.34)	17 (68%)	77.71 (12.15)	19 (79%)	NR	NR	NR	2 gm of sunflower oil		8 weeks

Two independent authors performed the quality assessment. The overall risk of bias of the included studies was high-risk or of some concern (Table 2).

**Table 2:** Risk of bias assessment

Study	Randomization process	Deviations from intended interventions	Missing outcome data	Measurement of outcomes	Selection of reported results	Overall risk of bias
Emamat et al. 2022 [16]	Low risk	Low risk	Low risk	Some concerns	Some concern	Some concerns
Farhangi et al. 2020 [14]	Low risk	Low risk	Low risk	High risk	Some concerns	High risk
Tavakoli-Rouzbehani et al. 2022 [15]	Low risk	Low risk	Low risk	Some concerns	Low risk	Some concerns

Outcomes

**Figure 2:**
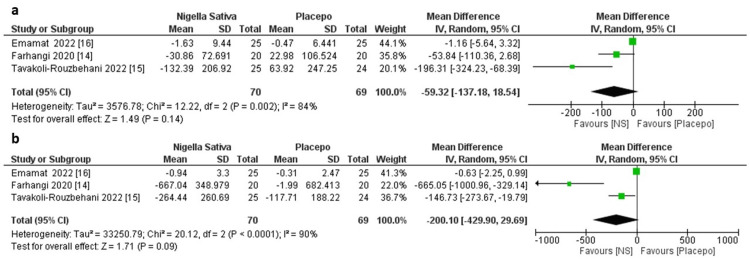
Forest plots of random-model MD of (a) the difference in the changes in ICAM between NS and control groups, and (b) the difference in the changes in VCAM between NS and control groups MD: mean difference; ICAM: intercellular adhesion molecule; NS: *Nigella sativa*; VCAM: vascular cell adhesion molecule; CI: confidence interval

Three studies were included in the analysis of the effect of NS on ICAM. The pooled random-effect size showed no difference between the NS group and the control group (MD = -59.32, 95% CI: -137.18 to 18.54; p = 0.14). The pooled data were severely heterogeneous (Tau² = 3576.78; Chi² = 12.22; p = 0.002; I² = 84%; Figure 2a). Likewise, three studies were included in the analysis of the effect of NS on VCAM. The pooled random-effect size showed no difference between the NS and control groups (MD = -200.1, 95% CI: -429.9 to 29.69; p = 0.09). The pooled data were severely heterogeneous (Tau² = 33250.79; Chi² = 20.12, p < 0.0001; I² = 90%; Figure 2b).

Discussion

Despite the considerable body of evidence underscoring the beneficial effects of NS supplementation on metabolic health risk factors, gaps remain in our comprehension of its potential impact on endothelial function. In an effort to bridge this knowledge gap, we conducted a systematic review and meta-analysis of RCTs to evaluate the effect of NS supplementation on endothelial function. This was specifically aimed at understanding its influence through the oxidative stress pathway by analyzing levels of ICAM-1 and VCAM-1. The findings from our meta-analysis indicate that NS supplementation does not exert a short-term effect on the concentrations of ICAM-1 and VCAM-1, suggesting that its beneficial impact on endothelial function, if present, may operate through mechanisms other than the modulation of these adhesion molecules in the short term.

NS has been extensively studied for its myriad health benefits, with research highlighting its anti-inflammatory properties [19], antioxidant effects, capability to lower blood glucose levels [19,20], and beneficial influence on lipid profiles across various diseases [21]. The volatile oil extracted from NS, particularly its principal component, thymoquinone, which constitutes 30% to 60% of the oil, is thought to play a pivotal role in mediating these health benefits [22]. Endothelial dysfunction, characterized by the impaired function of the cells lining the blood vessels, is regarded as an early indicator of atherosclerosis development, often identified by an increased expression of adhesion molecules such as ICAM-1 and VCAM-1 [23]. Recent evidence from a growing body of RCTs has suggested that NS administration significantly reduces ICAM-1 and VCAM-1 levels following two months of treatment [15,16]. However, our current meta-analysis indicates that NS supplementation does not lead to significant changes in the levels of ICAM-1 and VCAM-1, highlighting a discrepancy that underscores the complexity of NS's effects on endothelial function and the necessity for further detailed research.

The observed discrepancy between the findings from individual RCTs and those from the meta-analysis necessitates a more thorough investigation into several factors that could influence these results. The smaller sample sizes typically found in individual RCTs might contribute to a reduced statistical power, making it challenging to detect significant effects that a meta-analysis, which amalgamates data across multiple studies, is more equipped to reveal through enhanced statistical robustness. Moreover, variations in study designs, demographic characteristics of the populations studied, dosages, and the specific forms of NS utilized across different RCTs may lead to divergent outcomes. While a meta-analysis can smooth over these discrepancies by averaging outcomes across studies, this approach may also conceal specific effects that are more readily apparent in studies with more uniform populations or those that focus on particular aspects of NS supplementation.

The gender distribution in the included studies shows a predominance of male participants in the CAD studies (75-84%) and a predominance of female participants in the Hashimoto's thyroiditis study (85%). This gender imbalance may affect the generalizability of our findings. For instance, the physiological and hormonal differences between males and females could influence the response to NS supplementation. Therefore, future studies should aim to include more balanced gender representation to enhance the generalizability of the results.

NS may influence endothelial function through several alternative mechanisms beyond the modulation of ICAM-1 and VCAM-1. One notable pathway is its antioxidative capacity, primarily attributed to its bioactive component, thymoquinone [24]. Thymoquinone has been shown to enhance nitric oxide (NO) bioavailability by upregulating endothelial nitric oxide synthase (eNOS) activity, which is crucial for vasodilation and vascular health [25,26]. Additionally, NS exhibits anti-inflammatory properties by inhibiting nuclear factor kappa B (NF-κB) signaling, thereby reducing the expression of various pro-inflammatory cytokines such as tumor necrosis factor-alpha (TNF-α) and interleukins (IL-1, IL-6) [27]. These actions help mitigate oxidative stress and inflammation, both of which are key contributors to endothelial dysfunction. Moreover, NS has been observed to improve lipid profiles, decreasing total cholesterol and low-density lipoprotein (LDL) levels, which can reduce atherosclerotic plaque formation and promote endothelial integrity [28]. Finally, NS may exert antihypertensive effects by modulating calcium channels and reducing vascular smooth muscle contraction, thus improving overall vascular tone and endothelial function [12]. These multifaceted mechanisms suggest that NS could provide comprehensive vascular protection and improve endothelial function through a variety of biological pathways.

This systematic review and meta-analysis has several limitations that require careful consideration. First, the included studies display considerable variability in study designs, intervention protocols, and outcome measurements, particularly regarding the dosage and form of NS supplementation and the duration of interventions. This heterogeneity can significantly affect the comparability and consistency of the results. Additionally, the small sample sizes of all included studies potentially reduce the statistical power and limit the generalizability of the findings. Most studies were conducted in Iran and focused on specific populations, such as those with Hashimoto's thyroiditis, restricting the applicability of the findings to broader, more diverse populations. The relatively short follow-up duration, predominantly two months, may not be sufficient to evaluate the long-term effects of NS supplementation, potentially overlooking delayed outcomes or side effects. Furthermore, the quality of the included studies varies, with some lacking rigorous methodological approaches, impacting the strength and reliability of the evidence. Finally, there is a notable absence of detailed information on the adverse effects of NS supplementation, which limits our understanding of its safety profile.

Clinical implications

The findings of this meta-analysis highlight that NS supplementation does not significantly impact endothelial function, specifically in terms of ICAM and VCAM levels, in patients with CVD. This suggests that while NS may offer various health benefits, its role in modulating endothelial markers related to inflammation and atherosclerosis may be limited. Clinicians should consider these findings when recommending NS supplements for cardiovascular health, as current evidence does not support its efficacy in improving endothelial function through the reduction of ICAM and VCAM levels. Further research is needed to explore other potential mechanisms by which NS might benefit cardiovascular health and to establish its role within comprehensive CVD management strategies.

Future directions

Future research should aim to address the limitations identified in this meta-analysis, particularly the need for larger, well-designed RCTs with diverse populations and extended follow-up periods. Subgroup analysis and sensitivity analysis are crucial for future studies to better understand the differential effects of NS supplementation across various demographic and clinical subgroups, such as age, gender, and specific CVD conditions. Sensitivity analysis will help assess the robustness of the findings and determine the impact of different study characteristics on the results. Additionally, future studies should investigate the long-term effects of NS supplementation and explore other potential mechanisms of action, beyond the modulation of ICAM and VCAM, to fully elucidate its therapeutic potential in cardiovascular health.

## Conclusions

In conclusion, this systematic review and meta-analysis evaluated the effects of NS supplementation on endothelial function markers, including ICAM and VCAM. Based on the pooled data from three RCTs, the results did not demonstrate a statistically significant difference between the NS group and the control group in terms of ICAM or VCAM levels. However, it is important to note that the pooled data were severely heterogeneous, which may have impacted the results. Therefore, further high-quality studies with larger sample sizes and more standardized protocols are needed to confirm the potential therapeutic effects of NS on endothelial function and its associated inflammatory processes.
